# The Aggregation and Neurotoxicity of TDP-43 and Its ALS-Associated 25 kDa Fragment Are Differentially Affected by Molecular Chaperones in *Drosophila*

**DOI:** 10.1371/journal.pone.0031899

**Published:** 2012-02-22

**Authors:** Jenna M. Gregory, Teresa P. Barros, Sarah Meehan, Christopher M. Dobson, Leila M. Luheshi

**Affiliations:** 1 Department of Chemistry, University of Cambridge, Cambridge, United Kingdom; 2 Department of Genetics, University of Cambridge, Cambridge, United Kingdom; University Medical Center Groningen, University of Groningen, Netherlands

## Abstract

Almost all cases of sporadic amyotrophic lateral sclerosis (ALS), and some cases of the familial form, are characterised by the deposition of TDP-43, a member of a family of heteronuclear ribonucleoproteins (hnRNP). Although protein misfolding and deposition is thought to be a causative feature of many of the most prevalent neurodegenerative diseases, a link between TDP-43 aggregation and the dysfunction of motor neurons has yet to be established, despite many correlative neuropathological studies. We have investigated this relationship in the present study by probing the effect of altering TDP-43 aggregation behaviour *in vivo* by modulating the levels of molecular chaperones in a *Drosophila* model. More specifically, we quantify the effect of either pharmacological upregulation of the heat shock response or specific genetic upregulation of a small heat shock protein, CG14207, on the neurotoxicity of both TDP-43 and of its disease associated 25 kDa fragment (TDP-25) in a *Drosophila* model. Inhibition of the aggregation of TDP-43 by either method results in a partial reduction of its neurotoxic effects on both photoreceptor and motor neurons, whereas inhibition of the aggregation of TDP-25 results not only in a complete suppression of its toxicity but also its clearance from the brain in both neuronal subtypes studied. The results demonstrate, therefore, that aggregation plays a crucial role in mediating the neurotoxic effects of both full length and truncated TDP-43, and furthermore reveal that the *in vivo* propensity of these two proteins to aggregate and their susceptibility to molecular chaperone mediated clearance are quite distinct.

## Introduction

Although the ability to form intractable aggregates is an intrinsic property of all polypeptide chains, the formation of such species under physiological conditions is largely restricted to a subset of proteins associated with progressive pathological conditions [1], [2]. In particular, protein aggregation is a common feature of an increasingly prevalent group of neurological disorders that include Alzheimer's disease (AD) and Parkinson's disease (PD). A large body of genetic and pathological evidence indicates that the formation of such protein aggregates is a primary cause of neuronal dysfunction, and ultimately of neurodegeneration, in the case of both AD and PD. However, in almost all cases of sporadic and certain cases of inherited forms of amyotrophic lateral sclerosis (ALS) as well as a subtype of frontotemporal lobar degeneration with ubiquitin-positive inclusions (FTLD-U), such a link between the formation of protein aggregates and neurodegeneration remains to be established. Indeed, whilst both of these disorders are characterised by the formation of cytoplasmic and nuclear deposits of the protein TDP-43, they are also distinguished by its mislocalisation and a range of post-translational modifications including hyperphosyphorylation, ubiquitination and truncation, any one of which could result in loss of the normal function of TDP-43 as a regulator of transcription and splicing [3].

In order to investigate the molecular origins of ALS and related disorders, a number of cellular and animal models of TDP-43 expression have now been established. Whilst some of these models have indicated a toxic gain of function role for TDP-43 in protein aggregation and neurodegeneration, others have suggested that the cause of neuronal dysfunction is the loss of normal TDP-43 function, or indeed that the retention of the RNA binding activity of TDP-43 resulting in aberrant binding is a cause of its toxicity [4]–[6]. A further complicating factor in the interpretation of many of these studies is the need to account for the neurotoxicity not only of the full length TDP-43 protein, which could retain functional activity, but also of the non-functional N-terminally truncated 25 kDa fragment of TDP-43 that is found specifically in the brains of patients affected with ALS and FTLD-U [3].

In the present study we set out to investigate specifically the role of aggregation of full length TDP-43 and of an N-terminally truncated and disease-associated fragment, TDP-25, in causing neurodegeneration by upregulation of molecular chaperones, the inherent machinery of all biological systems that combats misfolding and aggregation; it is well established that molecular chaperones are able to inhibit the aggregation of proteins associated with neurodegenerative diseases [7], [8]. Furthermore, neuropathological studies, demonstrating the colocalisation of molecular chaperones with protein deposits in neurodegenerative diseases, and animal model studies, showing that they can inhibit protein aggregation and neurodegeneration *in vivo*, clearly indicate that these molecules play an important role in the regulation of pathological protein aggregation [9]–[11].

Many neurodegenerative disorders, including AD, PD and Huntington's disease (HD) have been modeled in *Drosophila melanogaster*, and these model systems have been formed to capture many of the key molecular features of these diseases [12]–[15]. Here, we describe the construction of a *Drosophila* model of TDP-43 proteinopathy and demonstrate that both pharmacological and genetic upregulation of chaperones enable a dissection of the distinct roles played by aggregation in neurodegeneration induced both by TDP-43 and its N-terminally truncated fragment TDP-25.

## Results

### A *Drosophila* model of TDP-43 proteinopathy

**Figure 1:**
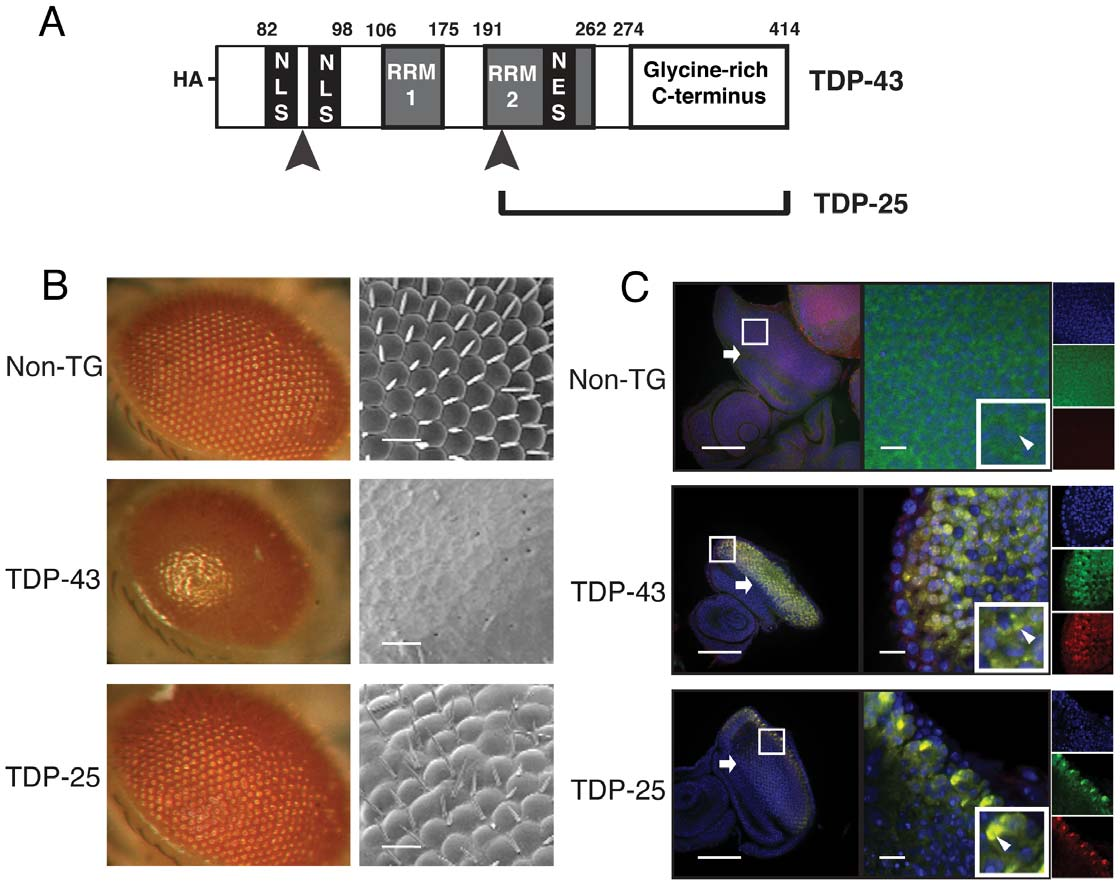
Neurotoxicity of TDP-43 expression in *Drosophila melanogaster*. A) Scheme of constructs used for expression. Both constructs have an HA-tagged N-terminus. The nuclear localisation sequence (NLS) [39] is indicated by two black rectangles between residues 82 and 98, the RNA-recognition motifs (RRM) [39] or RNA-binding domains (RRM 1, between residues 106 and 175, and RRM2, between 191 and 262) are indicated by two gray rectangles, and the nuclear export sequence (NES) is represented by the black rectangle between residues 239 and 250 within RRM 2. Arrowheads indicate caspase cleavage sites at residues 89 and 219 [40]. B) Optical and scanning electron microscope images demonstrating the effects of the TDP-43 and TDP-25 constructs when expression is driven in the photoreceptor neurons with *gmr-GAL4*. Colour pictures represent optical micrographs, taken at 7.5× magnification, of fly eyes and flies of the same genotype imaged using a scanning electron microscope at 200× magnification are shown in the adjacent picture. Scale bars represent 20 µm. C) Low and high magnification confocal microscopy images of imaginal eye discs of third instar larvae expressing TDP-43 or TDP-25 in the photoreceptors under the control of *gmr-GAL4*. TDP-43 and TDP-25 distribution is shown by anti-HA (red) staining, anti-ubiquitin (green) reveals ubiquitinated species, and TOTO-3 (blue) reveals nuclei. White arrowheads indicate aggregates, white arrows indicate the location of the morphogenetic furrow, and white boxes represent the area shown at higher magnification. Low magnification scale bars = 100 µm and high magnification scale bars = 10 µm.

In order to evaluate the aggregation and neurotoxicity of both full length TDP-43 and its disease associated, N-terminally truncated 25 kDa fragment (TDP-25), two lines of transgenic *Drosophila* were generated, each expressing one of these proteins. In the first line, the human TARDBP gene was introduced into the *Drosophila* genome using a modified UAS-*GAL4* vector [16], and in the second line a gene encoding the TDP-25 fragment that had been truncated at the putative caspase-3 cleavage site between residues 216–219 was introduced in to the *Drosophila* genome in the same way (Figure 1A) [17]. Both constructs were incorporated into identical genomic loci to ensure that each is transcribed in the flies at the same level and to allow quantitative comparisons to be made between their resulting phenotypes [18]. This method has been demonstrated previously to be an effective way of ensuring equalisation of transcription rates for different transgenes in *Drosophila* [19].

Expression of aggregation prone proteins associated with neurodegenerative diseases in the photoreceptors of *Drosophila* typically cause a characteristic ‘rough eye’ phenotype in which the normal linear array of ommatidia and sensory bristles become disordered, and in addition the eye loses its natural pigmentation to varying degrees that depend on the toxicity of the proteins expressed [20]; this phenotype can be visualised both by optical and scanning electron microscopy. Expression of TDP-43 and TDP-25 in this way using the *gmr-GAL4* driver was found to result in a rough eye phenotype in both cases, although its severity was observed to vary dramatically between the two genotypes. Thus, flies expressing TDP-43 have a strong rough eye phenotype, characterised by an almost complete absence of structured ommatidia when examined by either optical or scanning electron microscopy, whereas TDP-25 flies show only a weak phenotype that is only clearly evident on high magnification analysis using scanning electron microscopy (Figure 1B).

The rough eye phenotypes elicited by expression of the full length and the truncated TDP-43 constructs are both associated with microscopic signs of protein aggregation. Immunohistochemical analysis of the developing eye disc reveals the presence of TDP-43 both in the cytoplasm and in the nuclei of cells anterior to the morphogenetic furrow where *gmr* driven expression occurs (Figure 1C). Strikingly, the aggregates of both forms of TDP co-stained for ubiquitin, which is a feature of the deposits of TDP-43 found in ALS and FTLD-U brains (Figure 1C). Small puncta typical of protein aggregates are also visible. In marked contrast, similar analysis of TDP-25 flies indicates that there are predominantly individual large aggregate structures in the cell, found only in the most anterior region of the eye disc. The anterior region of the eye disc is where *gmr* driven protein expression has been activated for significantly longer than in more posterior cells closer to the morphogenetic furrow [21].

The strong correlation between the length of time for which TDP-25 has been expressed across the anterior-posterior axis of the eye disc and the extent to which it accumulates in cells along this axis, suggests that TDP-25 may be more susceptible to proteostatic regulation than TDP-43, which is distributed more uniformly along this axis. This conclusion is also consistent with the observation that the total number of aggregates and the intensity of staining of TDP-25 detectable in the eye disc by confocal microscopy is dramatically lower than that of TDP-43 (Figure 1C). Whilst these data are suggestive that aggregation could play a role in TDP-43 and TDP-25 induced neurodegeneration, we sought to test this association more directly by abrogating protein aggregation through chaperone upregulation and examining the effect on the toxic phenotypes observed for both proteins.

### 17-AAG treatment reduces aggregation of both TDP-43 and TDP-25

**Figure 2:**
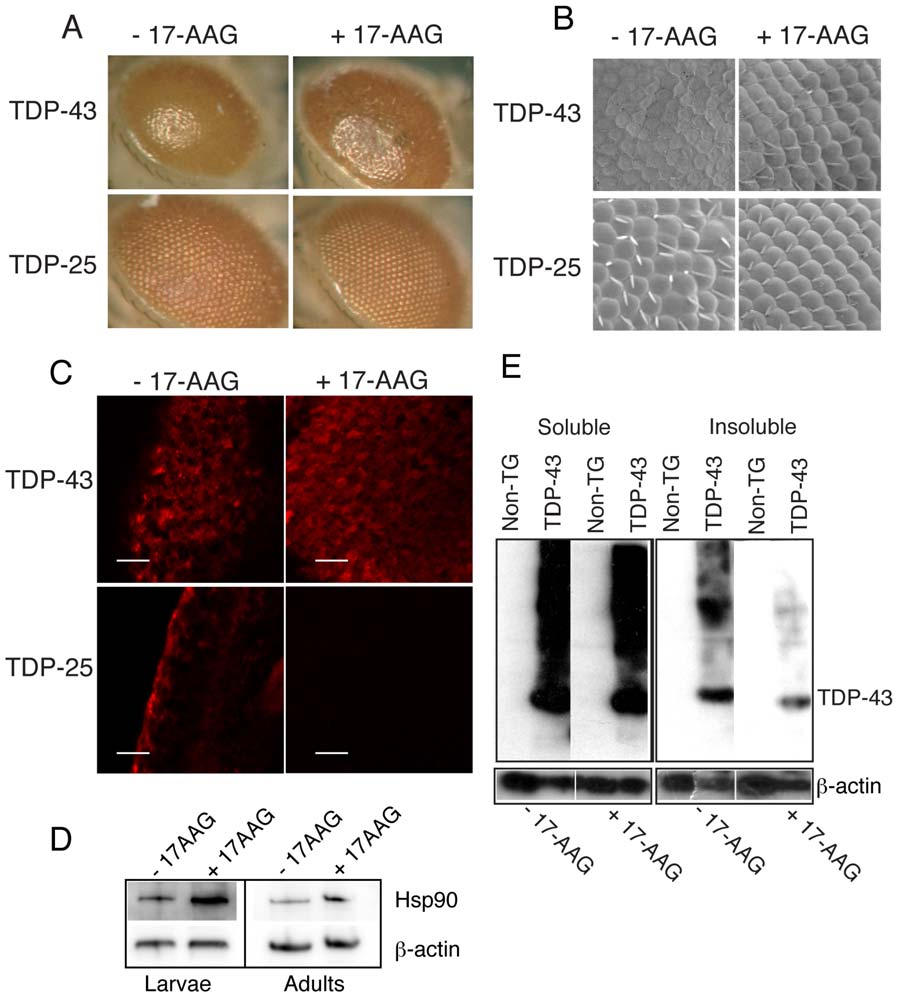
17-AAG treatment suppresses aggregation of TDP-43 and its disease associated fragment. A) Optical micrographs of flies expressing TDP-43 or TDP-25 treated with 17-AAG compared to untreated controls of the same genotype. B) Scanning electron microscope images of fly eyes taken at 200× magnification, scale bars represent 20 µm. C) Fluorescent confocal microscope images show the reduction in aggregation of TDP-43 and TDP-25 in the photoreceptors upon 17-AAG treatment compared to untreated flies of the same genotype. TDP isoforms are detected by anti HA staining (red) and nuclei by TOTO-3 staining (blue). D) Western blot revealing insoluble (9 M urea soluble) fractions of TDP-43 and TDP-25 from fly head homogenates in the presence or absence of 17-AAG, using an anti-HA antibody. β-actin was used as a loading control.

17-AAG, an analogue of the Hsp90 inhibitor geldanamycin, has been shown to up-regulate the heat shock response via activation of Hsf1, which itself regulates the transcription of a range of chaperones in *Drosophila*, and to abrogate the aggregation and toxicity of polyglutamine-containing proteins and α-synuclein in *Drosophila* and in cell cultures [22], [23]. We treated flies expressing TDP-43 and TDP-25 in their photoreceptors with 1.5 µM 17-AAG and observed the effects on the eye phenotypes described above. This treatment resulted in a partial rescue of the rough eye phenotype associated with TDP-43 expression (Figure 2A) as the eyes were of a more normal shape and size, were more intensely pigmented and had better defined ommatidia (Figure 2B), compared to those that were not treated. Nevertheless the eyes remained significantly dysmorphic when compared to non-transgenic controls. Immunohistochemical detection revealed that treatment with 17-AAG resulted in a significant reduction in the appearance of punctate aggregates of TDP-43, a redistribution of the protein into a more diffuse staining pattern throughout the cytoplasm and the nuclei of cells, and also the appearance of staining in more posterior cells within the eye disc (Figure 2C). This apparent inhibition of aggregation is supported by the results of SDS PAGE and western blot analysis in which the insoluble fractions of proteins extracted from fly heads were almost completely depleted of TDP-43 when flies had been treated with 17-AAG (Figure 2D).

The response of flies expressing TDP-25 to treatment with 17-AAG was observed to be quite distinct from that of TDP-43. In this case, the mild rough eye phenotype elicited by TDP-25 expression was completely rescued and, instead of causing a redistribution from punctate into more diffuse staining across a wider number of more posterior cells within the eye disc, as was observed for TDP-43, 17-AAG treatment results in the complete clearance of TDP-25 from the photoreceptor cells (Figure 2C). By contrast to western blot analysis of TDP-43, similar experiments performed on insoluble protein fractions extracted from flies expressing TDP-25, revealed that no protein could be detected by this method. This finding is also consistent with previous observations of a similar N-terminally truncated TDP-43 fragment in *Drosophila* in which expression could only be confirmed by RT-PCR [19].

### Chaperone upregulation reveals different relationships between aggregation and toxicity for TDP-43 and TDP-25

17-AAG is known to have pleiotropic effects on the regulation of multiple cellular processes, including autophagy and apoptosis, as well as inducing chaperone expression [22], [24], [25]. Thus, in order to explore whether aggregation could be modulated more specifically, we proceeded to examine the effects of genetic upregulation of the heat shock protein CG14207 that is intrinsic to *Drosophila* and has sequence homology with human αB-crystallin and human HspB8. This chaperone has been found previously in a genetic screen to reduce the neurotoxicity of the poly-glutamine containing protein ataxin 3 associated with spinocerebellar ataxia type 3 (SCA3), a disease that exhibits a similar cytoplasmic aggregation phenotype to the TDP-43 proteinopathies [26], [27]. In this previous study the authors established that the transgenic line EP 1348, used for the over-expression of CG14207, was indeed specifically activating the correct gene and not other transcription units close to the P-element insertion site. Due to the well-characterised nature of this chaperone line in *Drosophila*, we decided that CG14207 was an excellent candidate to establish whether chaperones can affect the aggregation and the subsequent toxicity of TDP-43 and TDP-25.

**Figure 3:**
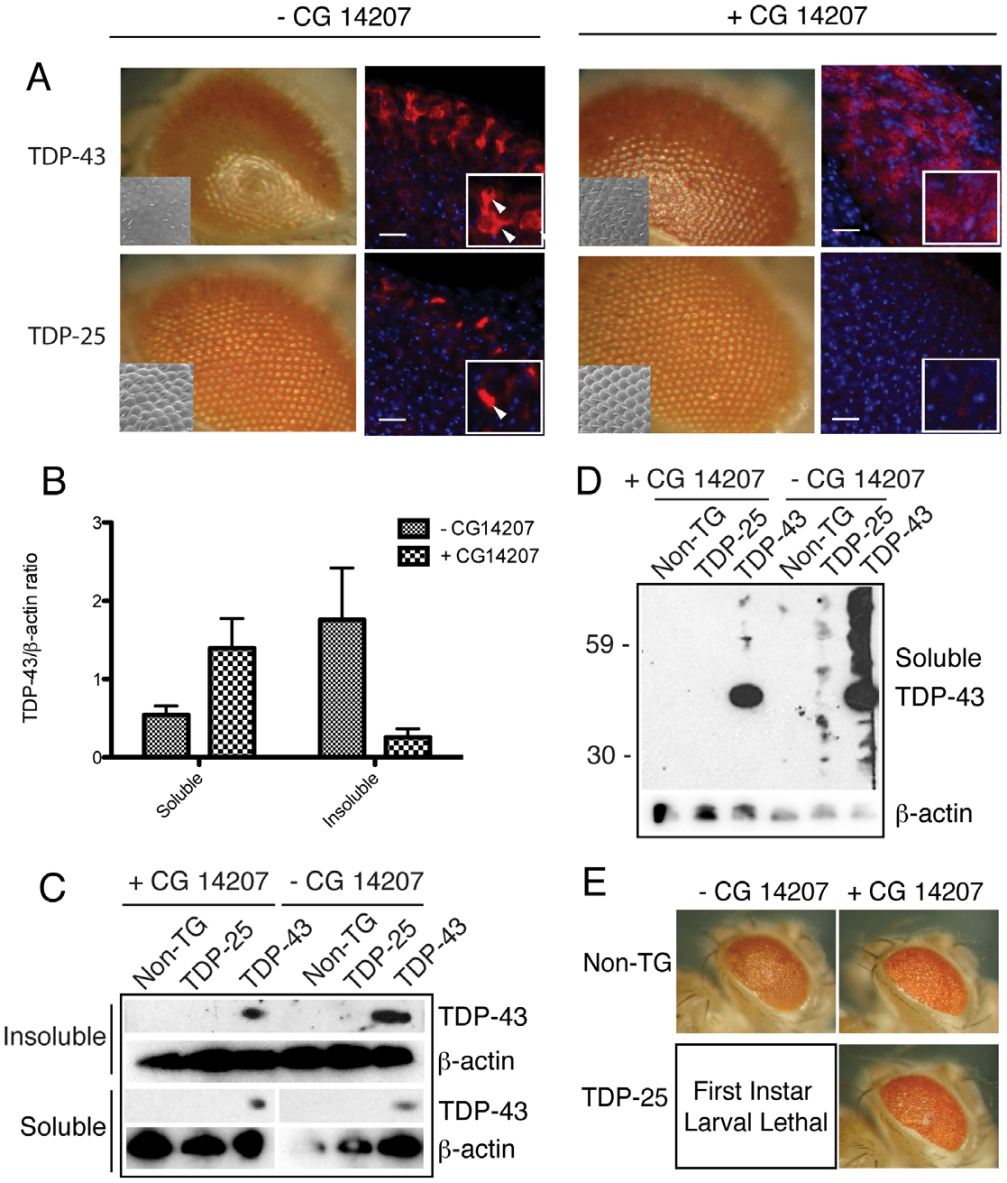
Genetic upregulation of an endogenous *Drosophila* chaperone reduces toxicity of TDP-43 and its disease associated fragment. A) Light and scanning electron microscope images demonstrating the effects of the TDP-43 and TDP-25 constructs when expression is driven by *gmr-GAL4*, in the presence and absence of co-expression of the chaperone CG14207. Light micrographs taken at 7.5× magnification, of fly eyes and inset are flies of the same genotype imaged using a scanning electron microscope at 200× magnification. Adjacent are low and high magnification confocal microscopy images of imaginal eye discs of third instar larvae. TDP-43 and TDP-25 distribution is shown by anti-HA (red) staining. TOTO-3 (blue) detects nuclei. White arrowheads indicate aggregates. White arrows indicate the location of the morphogenetic furrows and white boxes represent the areas shown at higher magnification. Low magnification scale bars = 100 µm and high magnification scale bars = 10 µm. B) Western blot detecting soluble (RIPA soluble) and insoluble (9 M urea soluble) fractions of TDP-43 and TDP-25 from fly brain homogenates in the presence or absence of the co-expression of CG14207, under reducing conditions. β-actin was used as a loading control. C) Western blot detecting soluble (RIPA soluble) fractions under non-reducing conditions, using an anti-HA antibody. β-actin was used as a loading control. D) Light microscope images demonstrating the effects of the TDP-25 construct when expression is driven by a different *gmr-GAL4* driver, which generates expression to a greater extent than that used previously, in the presence and absence of the co-expression of CG14207. Light micrographs of *Drosophila* eyes taken at 7× magnification.

Flies overexpressing CG14207 were crossed with those expressing TDP-43 and TDP-25, and the effects on their neurotoxic phenotypes were monitored. A very similar pattern of results was obtained to those observed with 17-AAG treatment, as CG14207 expression is only partially able to rescue the neurotoxic effect of TDP-43 on developing photoreceptors (Figure 3A), and redistribution of TDP-43 to more diffuse cytoplasmic and nuclear staining patterns is observed. The level of TDP-43 detected in the insoluble fraction of protein extracts from fly head homogenates, analysed by western blot and SDS PAGE, was also reduced almost two-fold. By contrast, the level of soluble TDP-43, either observed in immunostained eye discs or by western blot analysis following SDS PAGE under reducing conditions, is actually increased upon co-expression with CG14207, indicating that the inhibition of its aggregation is not associated with an increase in its clearance (Figure 3B). There is, however, a significant reduction in the amount of high molecular mass, oligomeric species in the soluble fraction under non-reducing conditions when CG14207 is present (Figure 3C), indicating that TDP-43 is either stabilised in its monomeric form or readily incorporated into insoluble aggregates.

In a similar series of experiments with flies expressing TDP-25, where the TDP-25 neurotoxic rough eye phenotype is mild in comparison to that elicited by TDP-43 expression, complete rescue upon CG14207 co-expression was observed. It was important to determine whether the difference in the extent of the rescue by CG14207 of TDP-43 and TDP-25 toxicity was indeed due to the two proteins having different intrinsic susceptibilities to CG14207 activity or is attributable to the apparently lower steady state concentration of TDP-25 present compared to TDP-43. To address this question a stronger *gmr-GAL4* driver was used, which was able to drive expression of TDP-25 to a much higher level than that achieved by the driver used in Figure 3a-c. Even when TDP-25 expression was increased in this way, resulting in larval lethality, it was still possible to observe a complete rescue of TDP-25 toxicity, as measured by a restoration in adult viability and eye morphology comparable to driver only controls, when CG14207 was co-expressed (Figure 3D). Furthermore we show that the rescue by CG 14207 does not simply reflect reduced expression of the transgene resulting from over-expression of another protein, as when we co-express TDP-43 with GFP, which has no chaperone activity, we see no rescue of the toxic phenotype (Figure S1). As observed for 17-AAG treatment, this rescue was accompanied by a complete clearance of the TDP-25 protein from the photoreceptor cells, as seen by immunohistochemistry and western blot analysis following SDS PAGE under non-reducing conditions. In addition, western blot analysis, under non-reducing conditions, shows that TDP-25 can be observed in the soluble fraction as bands of high molecular mass oligomers, but not as a monomer, and these species are completely cleared upon co-expression with CG14207 (Figure 3C). This striking difference in behaviour between TDP-43 and TDP-25 suggests that whilst TDP-25 can readily be degraded by the cell if it is prevented from aggregating by chaperones, TDP-43 remains substantially more resistant to clearance.

### Chaperone upregulation has differential effects on neuronal dysfunction when TDP-43 and TDP-25 are expressed in adult motor neurons

**Figure 4:**
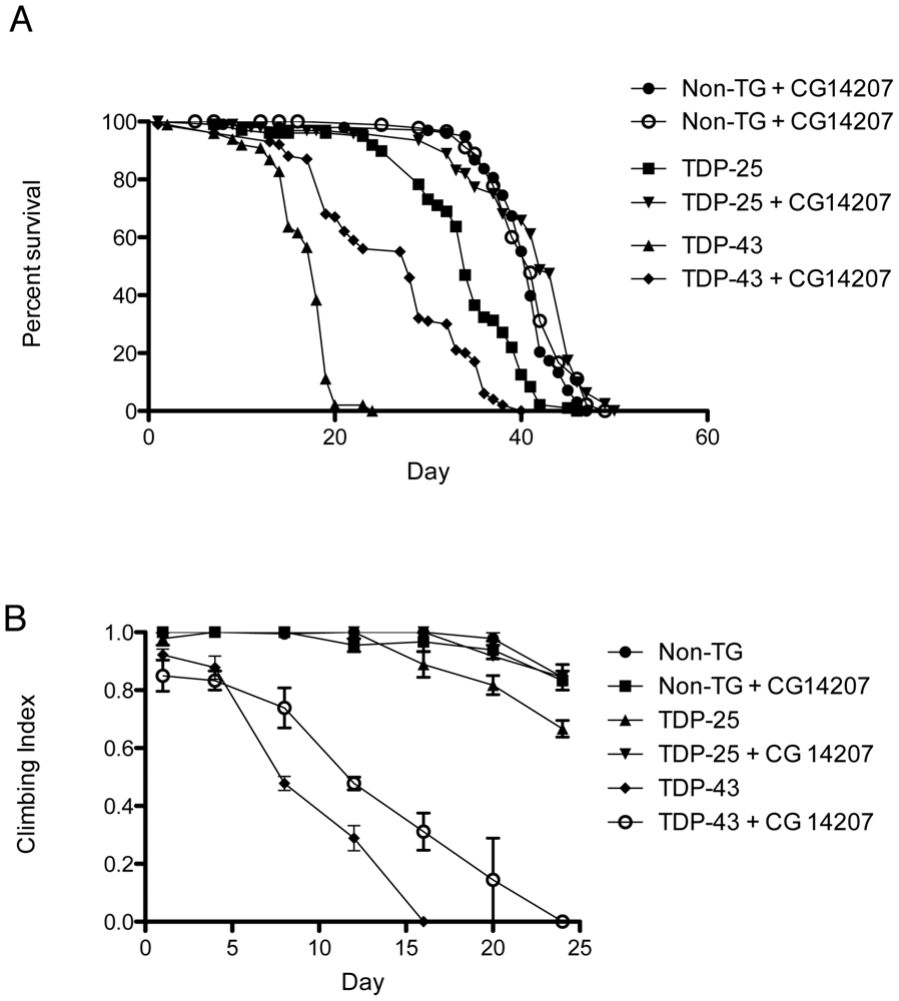
Chaperone upregulation has differential effects on neuronal dysfunction when TDP-43 is expressed in the adult motor neurons. A) Plot showing the Kaplan Meier survival data of TDP-43 and TDP-25 when expressed in adult motor neurons using a temperature sensitive Gal80 *D42*-*GAL4* driver compared to non-transgenic flies (WT). Lifespans were analysed by Kaplan-Meier statistics, n = 100 grouped into 10 tubes of 10 flies per genotype, differences between genotypes were analysed by Mann-Whitney U tests. B) Graph representing differences in motor function, analysed by climbing assay, when expressing TDP-43 and TDP-25 with and without CG14207 in adult motor neurons using the *Gal80-D42-GAL4* driver.

As ALS is a disease affecting the motor neurons, we examined the effects of the expression of TDP-43 and TDP-25 in motor neurons of adult flies (under the control of a heat-shock activated *D42-GAL4* driver). The median lifespans of the flies in which TDP-43 or TDP-25 expression was driven in these motor neurons were reduced by 57±2% and 17.7±3%, respectively, compared to control flies (Figure 4A; p<0.05). Co-expression of CG14207 with TDP-43, however, revealed a significant rescue in the toxic phenotype as flies show only a 28±6% reduction in median lifespan compared to control flies (p<0.05). By contrast flies co-expressing CG14207 with TDP-25 showed no significant difference in lifespan compared to wild type flies, indicating complete rescue. As the selective degeneration of upper and lower motor neurons is pathognomic for ALS, we also sought to investigate motor neuron function using a locomotor assay. From 8 days of age onwards flies expressing TDP-43 were observed to suffer a rapid decline in locomotor ability that is not observed in age matched non-transgenic flies (Figure 4B). Flies expressing TDP-25 were not, however, found to experience a significant decline relative to non-transgenic controls until 16 days of age, consistent with the relative toxicities observed when these two proteins are expressed in the eye. Co-expression of CG14207 with TDP-43 substantially reduces locomotor deficits compared to expression of TDP-43 alone, but the effect is only to reduce the rate of decline, with flies expressing CG14207 and TDP-43 still becoming immobile earlier than non-transgenic flies. Co-expression of CG14207 with TDP-25 not only reduced the locomotor deficit induced by TDP-25, but restored locomotor function to the same level as that measured for wild type flies at all ages measured.

**Figure 5:**
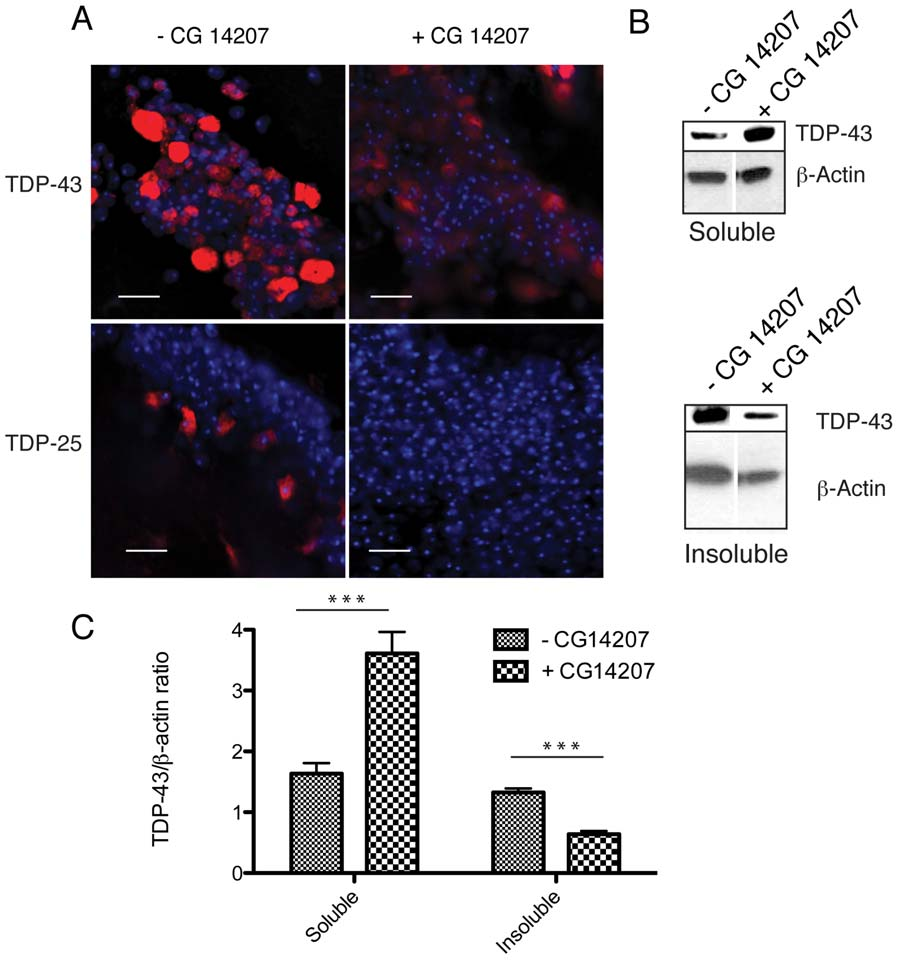
Chaperone upregulation reduces aggregation analysed by immunohistochemistry and SDS PAGE. A) Confocal microscope images showing the reduction in aggregation of all TDP isoforms in the adult motor neuron cell bodies in the 10-day-old brain upon co-expression with CG14207 compared to flies not expressing CG14207, 10 days after induction of expression. TDP isoforms are detected by anti-HA staining (red) and nuclei by TOTO-3 staining (blue). Scale bars = 10 µm. B) Western blot detection of RIPA soluble and urea soluble fractions of TDP-43 from fly head homogenates in the presence or absence of the co-expression of CG14207 under reducing conditions, using an anti-HA antibody. β-actin was used as a loading control.

Progressive diminution in the longevity and locomotor function of flies expressing TDP-43 with age is therefore associated with the accumulation of these proteins in the cytoplasm and in the nucleus. Immunohistochemical analysis of adult brains dissected from 10-day-old flies was carried out (Figure 5A), and co-expression of CG14207 was found to lead to a shift towards a more diffuse distribution of TDP-43 immunostaining within neurons. In order to examine whether or not this change in immunostaining reflects a change in the presence of aggregated forms of TDP-43, extracts from the brains of 10-day-old flies were analysed by SDS PAGE and western blotting (Figure 5B). In the absence of co-expression of CG14207, TDP-43 can be detected in both the detergent soluble and insoluble fractions, with the majority of protein being present in the insoluble fraction, indicating that it forms aggregates within the motor neurons in which it is expressed. Co-expression of CG14207 with TDP-43, however, results in a significant redistribution of the protein as the majority in this case was detected in the soluble fraction of fly head protein extracts. By contrast similar immunohistochemical analysis of 10-day-old flies expressing TDP-25 reveals that the ability of CG14207 to suppress completely the modest effect of TDP-25 on locomotor behaviour in the adult is associated with a complete clearance of TDP-25 from the neurons in which it is expressed, a finding consistent with our observations of TDP-25 toxicity in the eye.

## Discussion

Many cellular, invertebrate and vertebrate models of TDP-43 proteinopathy, based on the expression of full length, mutated, truncated or mislocalised protein, have been developed since the first reports emerged of the neuropathological and genetic role of this protein in FTLD-U, ALS and many other neurodegenerative diseases [5], [28]–[30]. Reports describing these models have variously suggested that motor or cortical neuron degeneration is correlated with the loss of endogenous TDP-43 from the nucleus [31], the presence of cytoplasmic or nuclear aggregates of full length or fragments of TDP-43 [4], [19], [32], [33], retention of the functional RNA binding domains of TDP-43 [4], [19] or the aggregation of the C-terminal fragment of TDP-43 [34]. However, variations in the construction of these models, and their reliance on making post hoc correlations between their histopathological characteristics and the loss of motor neurons, makes it very challenging to assess the significance of these different conclusions.

In the present study we set out to address directly the fundamental question of the role played by the aggregation of TDP-43 in its neurotoxicity by investigating the effects of increasing the intrinsic ability of cells to prevent protein aggregation by modulating molecular chaperones. This approach has allowed us to manipulate aggregation in a controlled manner and then to make quantitative comparisons of the neurotoxicity of TDP-43 with or without suppression of its aggregation. In order to achieve this objective we established a *Drosophila* model in which expression of TDP-43 and an ALS-associated 25 kDa fragment, TDP-25, can be driven in selected tissues so as to generate similar patterns of aggregation and neurodegeneration to those observed in FTLD and ALS patients.

The results suggest that the C-terminal fragment, TDP-25, which *in vitro* studies indicate is more aggregation prone than full length TDP-43 is, nevertheless, significantly less toxic than TDP-43 [35]. In addition, we find that steady state levels of TDP-25 are significantly lower than those observed for TDP-43 despite their transcription from identical genomic loci, suggesting that TDP-25 is cleared more efficiently than TDP-43, a finding consistent with observations from other studies in both *C. elegans* and *D. melanogaster* [19], [36]. This result provides an explanation for its reduced pathogenicity, which can be attributed to the fact that the truncation is likely to destabilise the protein, resulting in a disordered structure that is more susceptible to degradation. TDP-25 causes only a relatively late onset reduction in motor abilities when expressed in *Drosophila* motor neurons, and accelerates the normal decline in locomotor ability by only 10–15% in our model. Furthermore it causes only a mild rough eye phenotype compared to TDP-43. Moreover these impairments can be completely rescued by genetic upregulation of the chaperone CG14207, resulting in the complete clearance of TDP-25 aggregates even when the expression level of TDP-25 is increased by using a stronger *gmr* driver. This result indicates that the misfolding and aggregation of this fragment accounts completely for its neurotoxic effects. These results raise the issue of the extent to which the generation and aggregation of this fragment, which is specifically detected in patients suffering from FTLD-U or ALS, could be the cause of the underlying neurodegeneration observed in these diseases.

We also find that chaperone upregulation, and subsequent reductions in the microscopic and biochemical detection of aggregates of TDP-43, is associated with a reduction in its pathogenic effects demonstrated by photoreceptor degeneration or motor neuron dysfunction. In the case of flies expressing TDP-43, unlike those expressing TDP-25, this reduction in toxicity is incomplete, a finding consistent with the continued presence of aggregated TDP-43 that we have demonstrated biochemically by the detection of urea insoluble protein, albeit at much lower levels, in the brains of *Drosophila* when it is co-expressed with CG14207. This result is likely to reflect the inability of CG14207 expression, at the levels achieved by these experiments, to prevent or reverse completely the formation of toxic TDP-43 aggregates. Indeed it has been shown previously that other chaperones containing crystallin domains (homologous to those present in CG14207), such as α-crystallin and αB-crystallin may prevent the formation of insoluble, amyloid fibrils of other proteins but, depending on their stoichiometric ratios with respect to their client proteins, may also stabilise or even enhance the formation of relatively soluble but potentially neurotoxic non-fibrillar aggregates [37].

In conclusion we have exploited the upregulation of chaperones to probe the *in vivo* aggregation behaviour of TDP-43 and TDP-25, and its relationship with neurotoxicity. Our results demonstrate that whilst both TDP-43 and TDP-25 form aggregates *in vivo*, the abundance of these aggregates, the ease with which they can be prevented from forming by the small heat shock protein CG14207 and their neurotoxic effects are very substantially different. The important role of aggregation in the TDP-43 proteinopathies is therefore consistent with our understanding of other protein misfolding diseases such as AD, PD and HD.

## Methods

### Generation of transgenic *Drosophila* and fly stocks

HA-tagged TDP-43 constructs were codon optimised for expression in *D. melanogaster* and synthesised by Genscript (Piscataway, NJ, USA). Each construct was cloned into the multiple cloning site of pUAST-AttB and then subcloned into a modified pCasper vector (pCa4B, a kind gift of M. Markstein and N. Perrimon, Harvard University, USA). Differences in the expression of the constructs that could arise from their integration at different genomic loci were therefore eliminated as the pCa4B vector contains sites for exploiting the PhiC31 system for site-specific integration of transgenes. All injected constructs were incorporated at the same genomic locus (51D) on Chr. II (Bestgene Inc, USA) [18]. All other fly stocks used were obtained from Bloomington Stock Centre, Indiana, USA.

### Optical and scanning electron microscopy

TDP-43 and TDP-25 flies were crossed with *gmr-GAL4* flies (Bloomington Stock ID: 1104 & 8121). All flies were kept in a temperature and humidity controlled incubator maintained at 25°C, 70% humidity. Optical microscope pictures were taken of 1-day-old TDP/*gmr-GAL4* offspring at a magnification of 7.5×. SEM images were collected as described previously [12].

### Immunohistochemistry

Third-instar larval imaginal eye discs and adult brains were dissected, fixed in 4% PFA and blocked in 5% BSA in 0.05% PBS-Triton. This procedure was followed by incubation overnight with rat anti-HA-biotin high affinity antibody (Clone 3F10, Roche) and mouse anti-ubiquitin antibody (P4D1, Cell Signalling). Discs or brains were then incubated overnight in Streptavidin Alexa Fluor 594 conjugate (Invitrogen) and anti-mouse FITC, and counterstained with TOTO-3 (Invitrogen) to detect nucleic acids.

### Western blot analysis

Five flies per genotype were decapitated, homogenised in RIPA buffer and centrifuged to pellet the insoluble forms of proteins. The pellets were then resuspended in 9 M urea and centrifuged again to collect urea soluble protein. Each of the protein samples was separated on 4–12% Bis-Tris gel (Invitrogen) under reducing or non-reducing conditions and transferred to PVDF membranes (Millipore). Blots were blocked with 5% non-fat milk and then incubated with primary antibodies: anti-HA-biotin, high affinity (3F10) antibody (Roche) followed by incubation with anti-rat secondary antibody conjugated to HRP. Bands were detected using a SuperSignal® West Femto Maximum Sensitivity Substrate kit (Thermo Scientific). Loading controls using a mouse anti-β-actin antibody (Abcam) and anti-mouse HRP (Dako) were performed on all western blots.

### Geldanamycin treatment

The geldanamycin analogue 17-allylamino geldanamycin (17-AAG; Sigma) was resuspended in ethanol and diluted in distilled water to a concentration of 1.5 µM. 300 µL aliquots of drug were added to the surface of pre-made fly food and allowed to dry for 2 days. TDP-43 and TDP-25 flies were crossed with *gmr-GAL4* flies and maintained on either standard cornmeal-agar food treated either with distilled water or 1.5 µM 17-AAG to allow the larvae to ingest the drug. Microscopy, immunohistochemistry and western blot analyses were performed on drug treated and control offspring as described above.

### Longevity assay

Crosses were set up at 25°C. Non-virgin females were kept 10 per vial and moved to 29°C within 24 hours of eclosion and were transferred to fresh food and counted every 2–3 days. The experiments were performed in a temperature-controlled incubator at 29°C. Temperature sensitive Gal80 *D42-GAL4* is activated by heat shock and so simply moving flies from 25°C to 29°C induces expression in motor neurons. Median survivals for each vial were calculated using Kaplan-Meier survival statistics (n = 100 per experiment) and differences between genotypes were analysed using a Mann-Whitney U test (n = 10 tubes of 10 flies each).

### Climbing assay

Motor function was assessed by a climbing assay. Flies expressing TDP-43 and TDP-25 in motor neurons from the day of hatching, with and without CG14207, were generated in separate experiments as described above. 10 flies were placed into a single tube and analysed every second day after the initial heat shock; this procedure was repeated three times on the same tube of flies for each day. A climbing index score was calculated as described previously [38]. The average climbing index of the three replicates was calculated for each time point and plotted on a graph.

## Supporting Information

Figure S1 **Light microscope images demonstrating the effects of TDP-43 when expression is driven by** ***gmr-GAL4*** **, in the presence and absence of a non-toxic control protein (UAS-GFP).**
(TIF)
