# Supplementary figures and images for: The Aggregation and Neurotoxicity of TDP-43 and Its ALS-Associated 25 kDa Fragment Are Differentially Affected by Molecular Chaperones in Drosophila
Source: PLoS One. 2012 Feb 22;7(2):e31899. doi: 10.1371/journal.pone.0031899 (PMC3284513; doi:10.1371/journal.pone.0031899)

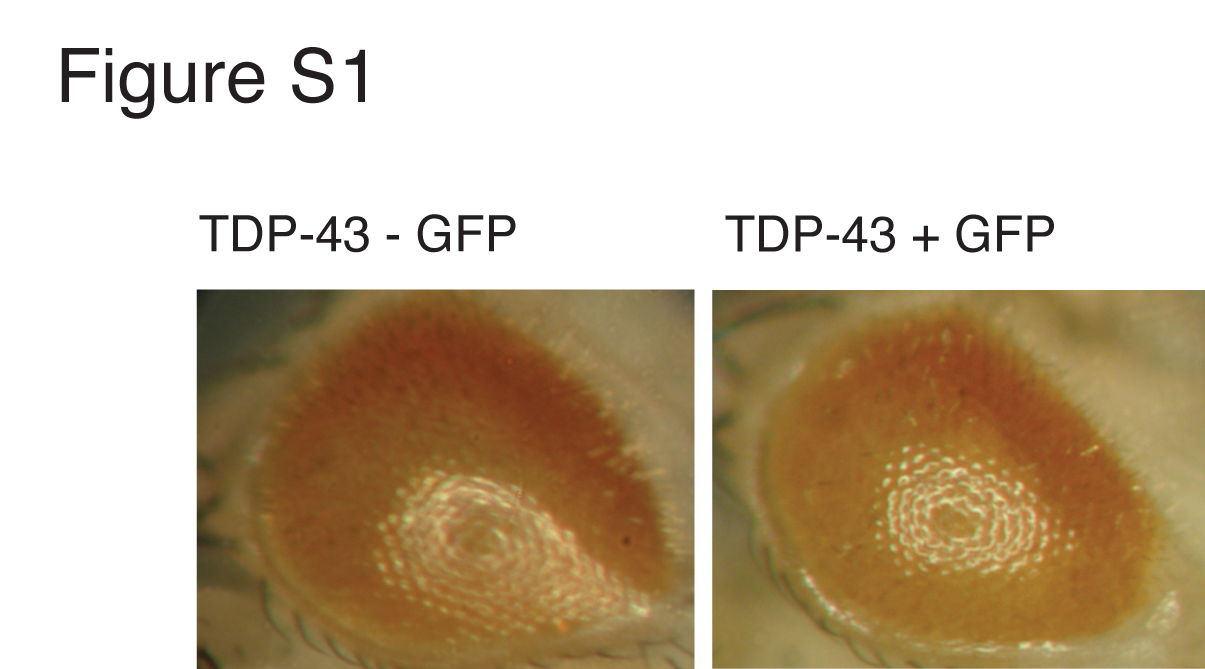

Supplement: Figure S1 — Light microscope images demonstrating the effects of TDP-43 when expression is driven by gmr-GAL4 , in the presence and absence of a non-toxic control protein (UAS-GFP). (TIF) [file pone.0031899.s001.tif]
